# Ups and Downs of Water Photodecolorization by Nanocomposite Polymer Nanofibers

**DOI:** 10.3390/nano9020250

**Published:** 2019-02-12

**Authors:** Shahin Homaeigohar, Niharika Krishna Botcha, Eman. S. Zarie, Mady Elbahri

**Affiliations:** 1 Nanochemistry and Nanoengineering, School of Chemical Engineering, Department of Chemistry and Materials Science, Aalto University, Kemistintie 1, 00076 Aalto, Finland; eman.zarie@aalto.fi; 2 Nanochemistry and Nanoengineering, Institute for Materials Science, Faculty of Engineering, University of Kiel, 24143 Kiel, Germany; bnkp888@yahoo.com; 3 Department of Therapeutic Chemistry, National Research Centre, Dokki 12311, Giza, Egypt

**Keywords:** water treatment, photocatalysis, dye removal, nanofiber, photodegradation

## Abstract

Given the exponentially expanding water pollution causing water scarcity, there is an urgent need for operative nanotechnological systems that can purify water, with insignificant energy consumption, and rapidly. Here, we introduce a nanocomposite system based on TiO_2_ nanoparticles (NPs) and PES nanofibers (NFs) that can adsorb and then photodecompose organic water pollutants such as dye molecules. We evaluate pros and cons of this system with respect to its purification efficiency and structural properties that can be impacted by the photocatalytic activity of the nanofillers. While the material is superhydrophilic and able to remove 95% methylene blue (MB) from water via adsorption/photodecomposition, its thermomechanical properties decline upon UV irradiation. However, these properties still remain at the level of the neat NFs. The removal behavior is modeled by the first- and second-order kinetic models from the kinetic point of view. The nanocomposite NFs’ removal behavior complies much better with the second-order kinetic model. Overall, such feedbacks implied that the nanocomposite can be effectively applied for water treatment and the structural properties are still as reliable as those of the neat counterpart.

## 1. Introduction

The textile dyeing and printing industries are two main sources of water pollution [1]. They release dyes, i.e. highly colored, low biodegradable organic compounds, into the water streams [2,3,4,5]. In fact, 1%–20% of the entire global production of dyes is released in the textile effluents [3,6] that can seriously harm the metabolism of living organisms including humans and that can adversely impact the environmental and natural processes like eutrophication [3,6].

**Figure 1:**
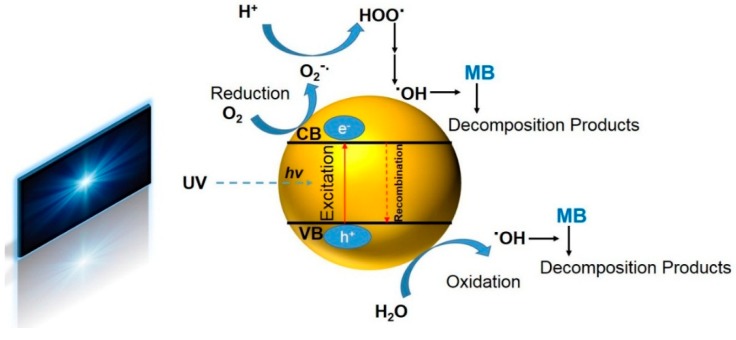
A schematic demonstration of the photocatalysis process of TiO_2_ leading to the decomposition of methylene blue (MB) (redrawn based on the schematic presented in Reference [12]).

Accordingly, there is a large demand for advanced water treatment technologies that remove dye pollutants from water economically and efficiently. The conventional relevant processes such as adsorption or coagulation merely collect the pollutants by switching them to other phases [7] but do not totally “eliminate” or “decompose” them. Other strategies such as sedimentation, filtration, chemical oxidation, and biotechnology are also unable to wholly remove the pollutants; they involve chemical reagents and high expenses, are lengthy, and produce toxic byproducts [8,9,10,11,12]. Advantageous over the mentioned techniques, photocatalytic decomposition of organic pollutants e.g., dyes, is an eco-friendly technique. This approach enables the complete removal of organic pollutants by the semiconductors that show photocatalytic activity when subjected to light irradiation [13]. There is a diverse range of photocatalysts studied for water treatment including CdS [14], SnO_2_ [15], WO_3_ [16], SiO_2_ [17], ZnO [18], and Fe_2_O_3_ [19], but the most investigated one is indeed TiO_2_ [20]. This photocatalyst is widely recognized for its high efficiency, inexpensiveness, physicochemical robustness, extensive availability, and noncorrosive being [12,21]. TiO_2_ nanoparticles (NPs) allow for the degradation of different organic pollutants including dyes. As Figure 1 shows, the exposure of TiO_2_ NPs to ultraviolet (UV) light excites the valence band (VB) electrons and transfers them to the conduction band (CB), thereby creating energized “holes” in the VB [12,22,23]. In the next step, the released free electrons react with oxygen and produce superoxide radical anions ($O_{2}^{•-}$). On the other hand, the energized holes oxidize water (H_2_O) or hydroxyl ion (OH^−^) and create hydroxyl radicals (•OH). The generated radicals are able to subsequently decompose the adjacent organic pollutants efficiently. Despite various merits, wastewater decontamination using TiO_2_ NPs is still in the lab scale and has not found any practical application due to several bottlenecks [12]. Insufficient adsorption capacity to hydrophobic pollutants, remarkable aggregation problems, and challenging separation and recovery of the TiO_2_ NPs are such problems that need to be addressed [24,25,26].

One solution for the abovementioned challenges could be the nanocomposite strategy. In particular, the hybridization of TiO_2_ NPs with polymer NFs not only maintains their high exposure to the surrounding medium due to a notable surface area of the NFs but also hampers their drastic agglomeration and facilitates their recovery. However, the immobilization of the photocatalytic NPs onto polymeric NFs is not straightforward due to their different polarity that favors the agglomeration of the inorganic fillers in the polymer matrix [27]. In this relevance, the physical blending of inorganic NPs with polymers is not an optimum strategy to make a homogenous dispersion for the subsequent processing, e.g., electrospinning and aggregation could hinder the process and clog the needle. Moreover, physical blending has shown inefficient in terms of surface immobilization of the inorganic NPs, thus lowering their surface activity towards the water pollutants [28]. To enhance the distribution mode of inorganic NPs in a polymer matrix and thus the efficient immobilization of the NPs, the following approaches have been proposed [27,29,30,31,32,33]: (1) In situ polymerization of a polymer monomer in adjacent to the surface-modified inorganic particles. In this case, however, the proper dispersion and long term aggregation resistance of the NPs are not guaranteed. (2) In situ generation of inorganic particles in an organic phase, i.e., bulk polymer, polymer solution, and monomer systems: In this method, called “sol-gel”, thanks to the in situ nucleation and growth of the particles inside the polymer matrix, they are confined and thus unable to aggregate. Other than the mentioned approaches, wherein the organic and inorganic components are together during the synthesis, functionalization, and processing steps, sputtering of the photocatalytic nanomaterials onto the polymeric NFs has been suggested as well. Alberti et al. [34] deposited TiO_2_ thin nanosheets on the polyethersulfone (PES) NFs via a direct current (DC) reactive sputtering technique. This process is not simple and involves a sophisticated set-up which is assumed to raise the preparation cost. In addition, the photocatalytic phase is as 2-/3-D sheets and not as fine as 0D NPs. Thus the reactive surface area is by no means comparable with the NP counterpart’s. Conclusively, to prepare photocatalytic nanocomposite NFs with a homogenous distribution of NPs on the surface of NFs, the sol-gel approach seems to be the most promising. This technique can assure the formation of very fine particle sizes and negligible aggregation mainly due to the in situ growth of the inorganic NPs in a limited space of polymer matrix. Accordingly, here, we created a sol-gel-based nanocomposite system comprising PES (a well-known polymeric membrane material) NFs coupled with TiO_2_ NPs. In our study, PES is chosen thanks to its notable thermal and chemical stability and also its durability [35]. Additionally, PES shows an isoelectric point of 2.4–3.1 [36,37]; thus under basic conditions, –OH groups cover the surface and interact with cationic dyes such as MB. Given the alkaline condition of the dyeing factories’ wastewater streams, PES with a hydroxylated surface at the basic aqueous media could be an attractive adsorbent material for cationic dyes. In the suggested nanocomposite system, this adsorption is accompanied with a photodecomposition process by TiO_2_ NPs when exposed to UV irradiation.

Despite the listed advantages, the UV exposure and the photocatalytic activity of the TiO_2_ NPs might adversely affect the structural properties of the polymer encompassing them. Thus, here, we conduct a research to fairly judge about the pros and cons of the developed nanocomposite system for a photocatalytic-based water purification. There are a couple of similar systems in the literature [34,38], but to the best of our knowledge, none of them has reported about the effects of the UV irradiated TiO_2_ NPs on the structural properties of such a photocatalytic system. Thus, in this study, we investigate the adsorption ability and photodecomposition efficiency of TiO_2_/PES nanocomposite NFs. In parallel, we track any alteration in structural properties of such a system when subjected to UV irradiation. This bi-faceted vision is a distinguishing characteristic of our study, providing insight into practical applications of photocatalytic systems for water treatment.

## 2. Materials and Methods

*Materials:* PES (Ultrason E6020P; M_w_ = 58,000 and density of 1.37 g/cm^3^) was purchased from BASF (Ludwigshafen, Germany). The solvents of N,N-dimethylacetamide (DMAc) and trifluoroacetic acid (TFA) were obtained from Merck (Darmstadt, Germany) and Aldrich Chemical Co. (Milwaukee, WI, USA), respectively. Tetra-*n*-butyl titanate (TBT) (titania precursor) and MB were purchased from Sigma-Aldrich (St. Louis, MO, USA).

*Synthesis of the nanocomposite*: A PES solution (20 wt.%) was made by dissolving PES flakes (1 g) in DMAc (4 g) and acidized with TFA (0.1 g). In parallel, the titanium precursor sol-solution was prepared by hydrolysis of TBT (0.2 g) with TFA (0.2 g) for 6 h at the molar ratio (TFA/TBT) of ≈4 and then mixed with the PES solution. The amount of TBT was precisely calculated to eventually have 5 and 8 wt.% of TiO_2_ in the nanocomposite NFs [39]. The obtained solution was stirred and thus homogenized overnight for the subsequent electrospinning step. The details of electrospinning can be found in Reference [39].

After electrospinning, to induce the in situ formation of the TiO_2_ NPs in/on the NFs, a hydrothermal treatment was carried out. The NFs were soaked into a hot water bath (75 °C) for 10 h to start and to proceed the condensation reaction of the TiO_2_ precursor, leading to the formation of the TiO_2_ NPs [27]. The as-made nanocomposite NFs were dried in air overnight and eventually annealed at 100 °C for 6 h to crystallize the sol-gel formed amorphous TiO_2_ as anatase.

*Morphological characterizations*: The morphology of the nanocomposite nanofibrous mats with respect to the NFs diameter and bead formation was assessed via scanning electron microscopy (SEM) (LEO 1550VP Gemini from Carl ZEISS, Jena, Germany). Transmission electron microscopy (TEM) (Tecnai G2 F20 field emission at an acceleration voltage of 200 kV from Philips, Amsterdam, The Netherlands) was utilized to characterize the morphology and distribution mode of the TiO_2_ NPs within the PES NFs.

*Chemical characterizations*: Chemical surface analysis of the TiO_2_/PES NFs was carried out by Attenuated Total Reflection Fourier Transform Infrared (ATR-FTIR) spectroscopy using a Bruker Equinox55 spectrometer (BRUKER Optik GmbH, Ettlingen, Germany). The static water contact angle of a 0.5 μL droplet on the TiO_2_/PES nanofibrous mats was determined using a contact angle analysis system (Kruess DSA 100, Hamburg, Germany). Additionally, to track the crystallization of the NPs at different stages before and after the thermal treatment, structural analyses of them were carried out at room temperature using an X-ray diffractometer (XRD3000TT, Agfa Gevaert (previously RICH. SEIFERT & Co GmbH), Mortsel, Belgium) with Cu–Kα radiation (λ = 0.1541 nm). Further, to verify the formation of the anatase crystalline structure via the applied thermal treatment, the TiO_2_/PES NFs were totally pyrolized at 500 °C in air and their XRD spectrum was recorded.

*Thermomechanical characterizations*: Differential thermal analysis (DSC) (Netzsch DSC 204 Phoenix, Bavaria, Germany) and thermal gravitational analysis (TGA) (Netzsch 209 TG, Selb, Germany) were applied to determine the glass transition (*T*_g_) and thermal decomposition temperature (*T*_d_) of the nanocomposite NFs. The *T*_d_ was defined as the temperature at 5% weight loss. The influence of the addition of TiO_2_ NPs to the PES NFs as well as the effect of their photocatalytic activity on the static and dynamic mechanical properties of the nanocomposites were evaluated via Dynamic Mechanical Analysis (DMA) and tensile tests. The frequency-dependant elastic moduli of the nanofibrous structures were measured by a dynamic mechanical analyser (RSA II, Rheometrics Co., Piscataway, NJ, USA) equipped with a tensile fixture with a frequency sweep from 0.005 to 100 rad/s using a deformation amplitude of 0.5%. The dimensions of the nanofibrous samples were 23 mm × 3.9 mm with a thickness of 70 μm. The tensile test was also performed by a tensile machine (Zwick/Roell Z020-20KN, Ulm, Germany) featuring a 20-N load-cell at ambient temperature. The cross-head speed was 2 mm/min and the gauge length was 20 mm. The tensile samples were, in fact, rectangular stripes with dimensions of 10 mm × 80 mm and as thick as 60 μm.

*MB degradation test*: Briefly, 1 g of the NFs from two groups of neat and nanocomposite (containing 8 wt.% TiO_2_) was immersed in 5 mL aqueous solutions of MB (4, 7, and 9 mg/L) basified (pH 10) using ammonium hydroxide. The solutions containing the NFs were irradiated by UV light (UV lamp: Labino 35W; Solna, Sweden) while stirred for three hours, and the solution samples (1 mL) were collected after each hour. The MB concentration at different stages and with various NF samples was analyzed by monitoring the solution’s absorbance at 664 nm, i.e., the MB characteristic absorbance peak [40] using UV-Vis spectrophotometer (Lambda 900, Perkin Elmer, Waltham, MC, USA) and based on the Beer–Lambert equation. Correlating the MB absorbance intensity to concentration, the dye removal percentage was calculated using the following Equation (1) [41,42,43,44]:

(1) $$RE\;\left(\%\right)=\frac{C_{i}-C_{t}}{C_{t}}\times 100\%$$

where *C_i_* and *C_t_* are the MB concentrations (mg/L) at onset and after a given time (i.e., 1, 2, and 3 h), respectively.

The degradation kinetics of MB by the nanocomposite NFs before and after UV-irradiation (i.e., the photocatalytic process) was analyzed using the pseudo first-order and second-order kinetic model, expressed as [2,45,46,47]:

(2) $$\ln\left(\frac{C_{i}}{C_{t}}\right)=k_{app}t$$

where *k*_app_ (min^−1^) is an apparent rate constant for the photocatalytic degradation of MB.

(3) $$\frac{t}{q}=\frac{1}{k_{2}q_{e}^{2}}+\frac{t}{q_{e}}$$

where *k*_2_ (min g/mg) is the second-order rate constant and *q* (mg/g) is the amount of MB adsorbed at time (*t*) that can be calculated as follows:

(4) $$q=\frac{\left(C_{i}-C_{t}\right)v_{sol}}{m}\times 10^{-3}$$

where *m* is the adsorbent mass used (g) and *V_sol_* is the solution volume (L). If there is a linear plot of *t/q* versus *t*, it can be said that the kinetic is of a second order and that *k_2_* and *q_e_* (i.e., the amounts of MB adsorbed at equilibrium) can be calculated from the intercept and slope of the plots.

The MB desorption analysis was performed for the neat and nanocomposite (containing 8 wt.% TiO_2_) NFs already exposed to an arbitrarily selected MB solution (4 mg/L). The NF samples holding the MB molecules not completely decomposed after UV-irradiation were immersed in an ethanol solution with different pHs of 10 (alkaline), 7 (neutral), and 3 (acidic) adjusted by the addition of acetic acid for 3 h. The concentration of the desorbed MB molecules in the solution was determined by UV-Vis spectrophotometer, and the desorption percentage was calculated considering the primary MB concentration as 100%.

## 3. Results

**Figure 2:**
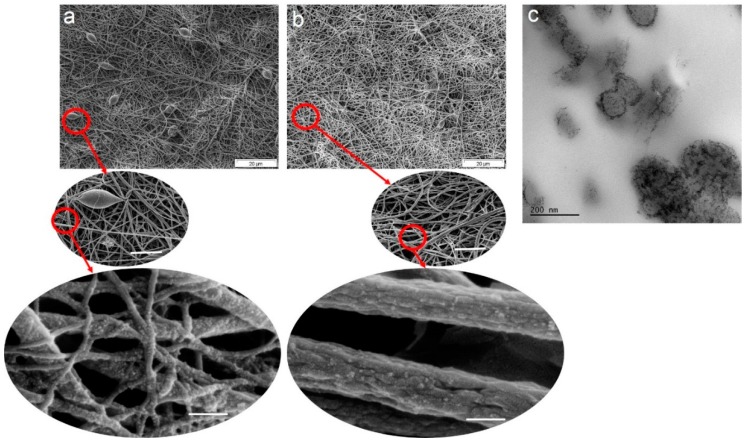
The morphology of the synthesized nanocomposite systems for water decolorization: The SEM images illustrate the morphology of the nanocomposite nanofibers (NFs) (containing 5 (**a**) and 8 wt.% TiO_2_ nanoparticles (NPs) (**b**), respectively) at three successive magnifications (the scale bars in the middle and lower images represent 5 and 0.2 µm, respectively). The highly magnified images in the lower row imply the decoration of the NFs by TiO_2_ NPs. (**c**) The TEM image shows how the TiO_2_ NPs are distributed within the cross section of the PES NFs.

Morphology, i.e., the uniformity of the NF mats in terms of porosity and absence of beads declining the exposed surface area, could be vital in their adsorption and photocatalytic performance. With respect to the formed TiO_2_ NPs, the location (interior or exterior), distribution mode, and size are of importance. In case the NPs are located on the exterior edge of the NFs, are homogenously distributed, and are as fine as possible, their photodegradation effect would be peaked. Figure 2 shows the morphology of the nanocomposite NF mats in two different TiO_2_ filling factors of 5 (Figure 2a) and 8 wt.% (Figure 2b) and at different magnifications. Apparently, the mat richer of the NPs is free of any beads and contains more uniform NFs in size. The presence of the TiO_2_ NPs (or in better words, their precursor) raises viscosity and viscoelastic force and lowers surface tension of the PES solution to be electrospun. As a result, the formation of structural irregularities such as beads declines, and the fiber diameter distribution becomes more uniform [28,48]. The TiO_2_ NPs are clearly visible on the body of the NFs. The formation and location of the NPs are also certified by TEM image (Figure 2c). The TEM image implies that the NPs are situated mostly at the outer edge of the NFs, though existing within the cross section as well. Such an observation is interpreted as the appropriate exposure of the NPs to the surrounding medium and particularly to the MB molecules. The NPs inside the NFs could be also responsible for an improved thermomechanical property, important for their industrial applications given that the material in practice will be under hot and stressful conditions.

**Figure 3:**
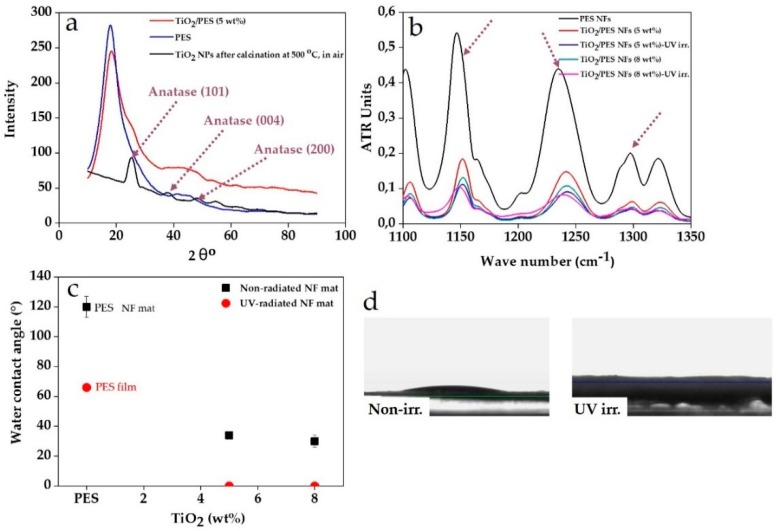
The crystallinity and surface chemistry of the nanocomposite NFs: (**a**) the XRD spectra for polyethersulfone (PES) NFs, TiO_2_ NPs (collected after the pyrolysis), and TiO_2_/PES NFs imply the emergence of the anatase characteristic peaks upon thermal treatment. (**b**) The ATR-FTIR spectra of TiO_2_/PES NFs in two different filling factors and as non- and UV-irradiated. (**c**) Water contact angle measurement results for TiO_2_/PES NFs in two different filling factors and as non- and UV-irradiated (the arrows mark the main characteristic peaks of PES). (**d**) Camera images compare the water contact angle on two samples of non- and UV-irradiated. Immediate absorption of water is evident for the UV-irradiated sample.

The crystallinity of the NPs and surface chemistry of the nanocomposite NFs play a major role in their adsorption/photocatalytic performance. The crystalline NPs show a larger surface energy and thereby an enhanced adsorption tendency [49]. XRD results, Figure 3a, shows that the PES NFs are totally amorphous and that their spectrum is featureless. In contrast, the TiO_2_/PES NFs feature a shoulder at 2θ = 25° representing the anatase (101) structure [50]. In general, the sol-gel-formed inorganic NPs are amorphous unless they undergo a thermal treatment. To prove that the peak (shoulder) has emerged due to the applied thermal treatment, the nanocomposite NFs were further heated up to 500 °C in air and their XRD spectrum was recorded. As seen in Figure 3a, the abovementioned anatase peak appears sharply, implying the improvement of the crystallization of the anatase phase. Also, the (101) peak becomes narrower, witnessing the enlargement of anatase crystallites [51]. Other than the (101) anatase peak, two other peaks emerge in the spectrum that represent the (004) and (200) anatase phases, respectively [50,51].

Figure 3b shows the ATR-FTIR spectra for the TiO_2_/PES NFs containing 5 and 8 wt.% TiO_2_ NPs before and after UV irradiation. The asymmetrical and symmetrical vibrations of the sulfone group of PES are represented by the absorption peaks at 1296 and 1146 cm^−1^, respectively. In addition, the absorption peak at 1234 cm^−1^ is attributed to the stretching vibration of the C–O–C bond in PES [52]. Compared to the PES NFs, the nanocomposite NFs’ characteristic peaks are weaker, implying coverage of the surface with TiO_2_ NPs. The characteristic peak of TiO_2_ should appear at 1103 cm^−1^ [53], overlapping with that of PES (1101 cm^−1^ i.e., the aromatic ring [35]), thus not recognizable separately. Moreover, the peaks are slightly shifted, representing the formation of a hydrogen bonding between TiO_2_ NPs and PES chains. The peak emerged at 1234 cm^−1^ for the PES NFs shift to 1239 and 1241 cm^−1^ for the TiO_2_/PES NFs with 5 and 8 wt.% TiO_2_, respectively. This shift takes place because of in situ formation of TiO_2_ NPs in the PES matrix [54] and because of hydrogen bonding between Ti–OH or Ti–OCH_2_CH_2_CH_2_CH_3_ and ether C–O–C bond (or sulfone SO_2_ group) during the hydrolysis and condensation of TBT forming into TiO_2_ sol [54]. UV irradiation also declines the intensity of the peaks more notably, implying photodegradation of the polymer matrix induced by the photocatalytic activity of the NPs. This effect is more evident for the nanocomposite NFs with a higher amount of TiO_2_ NPs.

The surface decoration of the PES NFs with TiO_2_ NPs can bring about a hydrophilicity effect and minimizes the wetting time of the structure, beneficial for water treatment processes. Figure 3c shows that the water contact angle of the NF samples notably declines by the presence of TiO_2_ NPs on the surface. It is worthy to note that in the case of the PES NFs, the contact angle is significantly larger than their film shaped counterpart, mainly due to the remarkable roughness and presence of air pockets between the NFs that maximize hydrophobicity, as explained by the Cassie–Baxter model [55]. The larger the amount of the surface resided NPs is, the lower water contact angle will be. Interestingly, the UV-irradiated NFs demonstrate a superhydrophilicity effect (0° contact angle) versus the non-irradiated ones, as witnessed by camera images (Figure 3d). This behavior can be attributed to the formation of an electron–hole pair in the VB and CB of TiO_2_ by UV-irradiation, reacting with H_2_O and O_2_ molecules in the proximity of the TiO_2_ surface. This interaction leads to the generation of peroxide intermediates that can react with the TiO_2_ surface and make Ti–O–H bonds, maximizing the hydrophilicity of the TiO_2_ NP’s surface [56].

**Figure 4:**
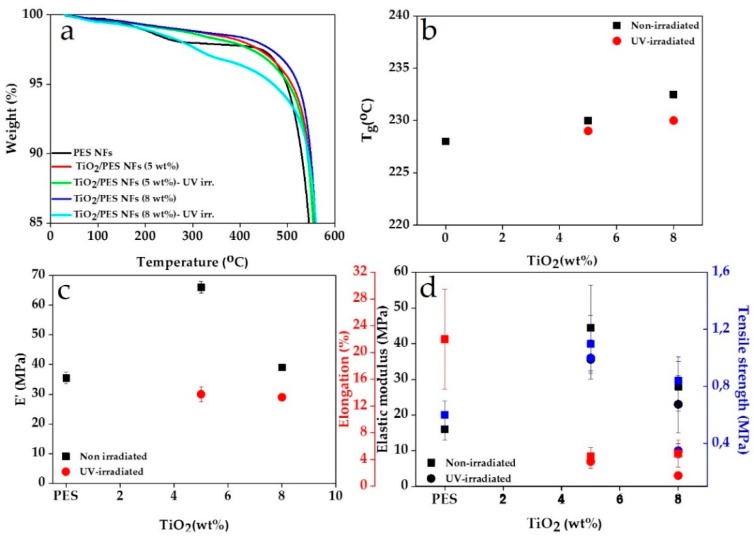
The thermomechanical properties of the nanocomposite NFs: (**a**) The thermogravimetric analysis (TGA) graphs imply an increase in the thermal stability of the NFs by the inclusion of TiO_2_ NPs but with a less effect for the UV-irradiated NFs. (**b**) The glass transition temperature rises for the nanocomposite NFs versus their neat counterpart. The increment in T_g_ is less notable for the UV-irradiated NFs. (**c**) The dynamic thermomechanical (DMTA) analysis witnesses the enhancement of storage modulus for the nanocomposite NFs, as far as they are not UV-irradiated. (**d**) The tensile test results imply higher mechanical properties, represented by elastic modulus and tensile strength and, on the other hand, the brittleness of the nanocomposite NFs. UV-irradiation, again, has a slightly detrimental effect on such properties.

While the surface residence of the TiO_2_ NPs guarantees a superhydrophilicity effect, those located within the NFs can affect the thermomechanical properties of the nanocomposite NFs. Figure 4a,b shows the thermal properties of the NFs with and without the presence of the TiO_2_ NPs and before and after UV irradiation. TGA results (Figure 4a) imply that the nanocomposite NFs are thermally more stable than the PES NFs, and this enhancement in thermal stability is proportional to the TiO_2_ NPs filling factor. However, UV-irradiation can lower this thermal stability down to the level of PES NFs and even lower (at 8 wt.%). These observations can be quantified as shown in Table 1. It is assumed that the electron/hole pairs formed in the CB and VB, respectively, react with O_2_ and thereby form a variety of active oxygen species including O_2_^−^, ^1^O_2_, .O_2_H, and .OH [57]. Subsequently, such active oxygen species initiate the degradation process when attacking the adjacent polymer chains. The degradation process is not limited to the surface and involves the polymer bulk and deeper regions when the active oxygen species penetrate into the material. Upon diffusion of the carbon-centered radicals into the polymer chain, their consecutive reactions end up with the chain scission with the oxygen incorporation and CO_2_ release [57]. As seen in Table 1, the larger the TiO_2_ NPs filling factor, the higher the UV absorption and thus more weight loss, i.e., photooxidative degradation, will be. The recorded weight loss certifies that some degradation products have been released in gaseous form [53]. With respect to the 5 wt.% TiO_2_ incorporated nanocomposite NFs, it seems that there is a balance between the reinforcing effect of the NPs and the weakening effect of their photocatalytic activity.

Figure 4b also stresses that the incorporation of TiO_2_ NPs into PES NFs can bring about a higher glass transition temperature thanks to the hydrogen bonding between the NPs and polymer chains as previously discussed based on the ATR-FTIR results. On the other hand, the photodegradation of the PES NFs after UV irradiation lowers T_g_ implying a chain scission and a higher mobility of low molecular weight chains upon heating. However, still T_g_ for the UV-irradiated nanocomposite NFs is comparable or even higher than that of PES NFs.

Figure 4c,d shows how the nanocomposite NFs behave mechanically particularly after UV irradiation. Once again, given the bonding of the nanoparticles to the polymer chains, the mechanical properties, statically and dynamically, are improved by the addition of the TiO_2_ NPs. A robust nanoparticle–polymer interface can guarantee an enhanced load transfer and thus higher mechanical properties [58,59]. As shown in Figure 4c, the dynamic storage modulus (*E*′) of the nanocomposite NFs is higher than that of the PES NFs, particularly at a 5 wt.% TiO_2_ filling factor. This behavior is the case when the NFs are subjected to uniaxial tensile stresses (Figure 4d). Elastic modulus and tensile strength rise for the nanocomposite NFs, but elongation declines, i.e., the NFs are becoming brittle. Again, the nanocomposite NFs with a 5 wt.% TiO_2_ filling factor show the best mechanical performance, probably due to a more homogenous distribution of the NPs within the NFs. Also, Luo et al. [54] state that the increase of the free volume of the polymer matrix around the TiO_2_ NPs can result in a loss of the mechanical properties. Regardless of the type of mechanical tests, the UV-irradiated nanocomposite NFs possess inferior mechanical properties versus their non-irradiated counterparts due to their photodegradation leading to main-chain scission or side-group abstraction. The chain scission, as mentioned earlier, can shorten the chain length and enhance the mobility of polymer chains, thus weakening mechanical properties [60]. However, compared to PES NFs, this group of nanocomposite NFs shows a similar dynamic mechanical performance, as witnessed by the DMA results (Figure 4c). Interestingly, in the case of tensile test, the UV-irradiated nanocomposite NFs mostly offer even better mechanical properties than PES NFs reflected in their higher tensile strength and elastic modulus. Conclusively, the photocatalytic activity of the nanofillers declines or neutralizes their reinforcing effect but does not worsen the structural properties of the nanocomposite NFs.

**Figure 5:**
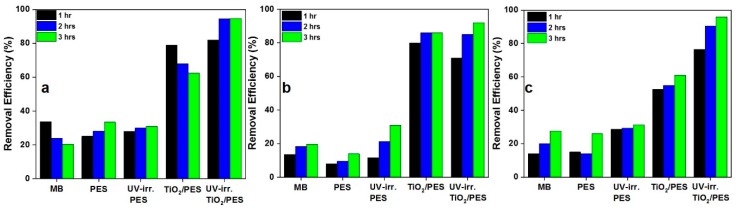
The adsorption and photodegradation of dye molecules by the nanocomposite NFs: the MB removal efficiencies of the PES and TiO_2_/PES (8 wt.%) nanofibrous adsorbents under alkaline condition before and after UV-irradiation for the MB aqueous solutions with concentrations of (**a**) 4, (**b**) 7, and (**c**) 9 mg·L^−1^.

To characterize the MB degradation efficiency of the nanocomposite NFs, we considered only the NFs with the highest content of TiO_2_ NPs (i.e., 8 wt.%) and compared their MB removal efficiency with that of the PES NFs. The experiments were carried out based on the MB aqueous solutions containing 4, 7, and 9 mg·L^−1^ MB under alkaline conditions (pH 10) which is typical to industrial dyeing wastewater streams. Figure 5 compares the MB removal efficiency of the nanocomposite NFs before and after UV irradiation with that of the control PES NFs. These results shed light on the adsorption and then the photodecomposition of MB pollutants by the nanocomposite NFs. In general, the nanocomposite NFs offer a notably higher removal efficiency before and after UV irradiation versus the PES NFs. In most cases, there is an increasing trend for the MB removal efficiency with time. After 3 h, the photodecomposition efficiency can reach up to 95% for the nanocomposite NFs (at 9 mg·L^−1^ MB concentration) while their highest adsorption efficiency is recorded as high as 86% (at 7 mg·L^−1^ MB concentration). In contrast, the PES NFs are able to show only 31% photodecomposition efficiency (at all the MB concentrations) and 33.5% adsorption efficiency (at 4 mg·L^−1^ MB concentration) after 3 h. The PES NFs adsorb the cationic MB molecules up to 33.5% due to their low isoelectric point of 2.4–3.1, enabling them to have a negatively charged, hydroxylated surface at alkaline conditions [2]. The generally poor removal efficiency of the PES NFs after UV irradiation is not out of imagination due to the absence of the photocatalytic material, i.e., TiO_2_ on their surface. However, a slightly enhanced removal efficiency seen for the UV-irradiated PES NFs versus the non-irradiated ones can be attributed to the formation of the radicals stemming from the photooxidation of PES, particularly the diphenylethersulfone site, caused by the UV induced chain scissions and the cleavage of the aromatic rings. Subsequently, MB is oxidized in the presence of such radicals [34,61,62]. If only the adsorption efficiency of the nanocomposite NFs is compared with that of the PES NFs, the existence of the TiO_2_ NPs plays a determining role and adsorbs MB molecules largely. Under alkaline conditions, the TiO_2_ NPs with the isoelectric point of 5.8 [63] are negatively charged and thus show a high electrostatic affinity towards the cationic MB molecules. The adsorbed MB molecules as well as those near the NPs inside the solution will undergo a photocatalytic degradation upon UV irradiation. Thus, obviously the UV-irradiated nanocomposite NFs show a higher MB removal efficiency than those that are non-irradiated.

The UV-induced photocatalysis by TiO_2_ (Figure 1) leads to the oxidative degradation of the MB dye, as the nearly complete mineralizations of C and of N and S heteroatoms into CO_2_, NH_4_^+^, NO_3_^−^ and SO_4_^2−^ respectively. Sequentially, this process includes the following sub-reactions [62]:

(1) TiO_2_ receives an efficient photon energy (*hv* ≥ 3.2 eV).
  (5) $$\left(TiO_{2}\right)+hv\to e_{CB}^{-}+h_{VB}^{+}$$
(2) Oxygen ionosorption takes place whereby oxygen is reduced, i.e., oxygen’s oxidation degree declines from 0 to −1/2.
  (6) $${\left(O_{2}\right)}_{ads}+e_{CB}^{-}\to O_{2}^{•-}$$
(3) Hydroxide ions are neutralized by photoholes, and thereby OH^•^ radicals are formed.
  (7) $${\left(H_{2}O↔H^{+}+OH^{-}\right)}_{ads}+h_{VB}^{+}\to H^{+}+OH^{•}$$
(4) Protons neutralize $O_{2}^{•-}$.
  (8) $$O_{2}^{•-}+H^{+}\to HO_{2}^{•}$$
(5) Temporarily, H_2_O_2_ is produced, and the dismutation of oxygen occurs.
  (9) $$2HO_{2}^{•}\to H_{2}O_{2}+O_{2}$$
(6) H_2_O_2_ is dissociated, and oxygen is reduced for the second time.
  (10) $$H_{2}O_{2}+e^{-}\to OH^{•}+OH^{-}$$
(7) The MB molecules (or any other organic material in the adjacent of TiO_2_; R) are oxidized through consecutive attacks by $OH^{•}$ radicals.
  (11) $$R+OH^{•}\to R^{•}+H_{2}O$$
(8) The MB molecules (R) can be directly oxidized by the reaction with holes.
  (12) $$R+h^{+}\to R^{+•}\to decomposition\;products$$

For instance, holes can react with carboxylic groups of an organic compound and, thereby, produce CO_2_.

(13) $$RCOO^{-}+h^{+}\to R^{•}+CO_{2}$$

Other than the generation of CO_2_, nitrogen and sulfur heteroatoms are also converted to inorganic ions such as nitrate, ammonium, and sulfate ions, respectively. In fact, the first step of MB photodecomposition is the bond cleavage of the C–S^+^=C functional group by OH^•^ that gives rise to the formation of sulfoxide, a sulfone, and a sulfonic group respectively, as recorded in the Fenamiphos degradation process [62]. The sulfoxide group reacts with OH^•^ and generates sulfone that is subsequently attacked by another OH^•^ and produces a sulfonic acid. Sulfonic acid is dissociated by another OH^•^ attack and releases SO_4_^2−^. With respect to carbon, the mineralization of organic carbon takes place by total organic carbon (TOC) removal and the generation of CO_2_ mainly via photocatalysis. As Houas et al. [62] report, the oxidative mineralization of MB is accompanied with chemical oxygen demand (COD) disappearance as well. At this point, a large fraction of MB vanishes, attributed to the opening of the aromatic rings, the exposure of carboxylic acid groups, and then the generation of CO_2_ according to the “photo-Kolbe” reaction (13). MB contains three N-based groups including the imino group located in the center of the molecule and two symmetrical dimethyl-phenyl-amino groups. The imino group undertakes a N=C cleavage under the influence of the cleavage of the double bond of −S^+^= in the para position of the central aromatic group. Subsequently, the amino group is replaced by an OH^•^ leading to the generation of phenol and the release of $NH_{2}^{•}$ that can further produce ammonia and ammonium ions. The latter compound is gradually oxidized to nitrate or hydroxylamine then nitrate. The two symmetrical dimethyl-phenyl-amino groups are degraded in a step-wise manner via the oxidation of one methyl group by OH^•^, thus generating an alcohol and then an aldehyde. The transient product is later oxidized into acid that is decarboxylated into CO_2_, following the photo-Kolbe reaction [62].

**Figure 6:**
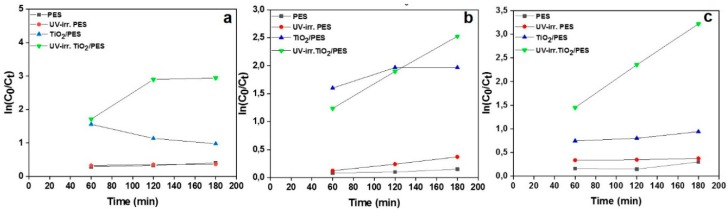
The first-order kinetic plots for the photocatalytic degradation of MB by PES and TiO_2_/PES (8 wt.%) NF adsorbents before and after UV-irradiation. The first-order kinetic plots are related to the MB aqueous solutions with concentrations of (**a**) 4, (**b**) 7, and (**c**) 9 mg·L^−1^.

**Figure 7:**
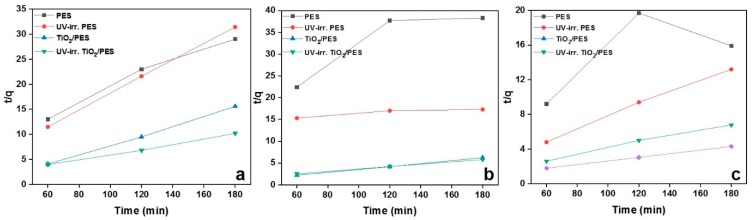
The second-order kinetic plots for the photocatalytic degradation of MB by PES and TiO_2_/PES (8 wt.%) NF adsorbents before and after UV-irradiation. The second-order kinetic plots are related to the MB aqueous solutions with concentrations of (**a**) 4, (**b**) 7, and (**c**) 9 mg·L^−1^.

In terms of kinetics, we fitted the obtained data with the pseudo first- and second-order models to acquire a better understanding of the removal process. First, the kinetic data were fitted to the first-order kinetic model (Equation (2)). Figure 6a–c shows the time dependency of *ln(C_0_/C_t_*) for the NF samples at different MB concentrations. From the linear plots, the removal rate constants (*K_app_*) can be calculated, as reported in Table 2. As seen in the Table, a *R^2^* value approaching 1 indicates a proper harmony with the first-order kinetic model, which is the case only for the UV-irradiated NFs immersed in the aqueous solutions containing 4 and 9 mg·L^−1^ MB. For the other conditions in terms of MB concentration and adsorbent’s composition, the kinetic data were further modeled with the pseudo second-order kinetic equation. Figure 7a–c shows the linear plots of *t/q* vs. *t* at different MB concentrations. From such plots, values such as *k_2_* and *q_e_* could be extracted (Table 3) that offer precious information regarding the removal kinetics and equilibrium capacity of the NF systems. As seen in Table 3, the *R^2^* values for the second-order kinetic model are mostly larger than 0.99, implying a good agreement of the experimental data with the second-order kinetic model for different samples excluding PES NFs. Therefore, the nanocomposite NFs’ removal behavior complies well with the second-order kinetic model. Based on this finding, the rate limiting step may be chemisorption, involving the valency forces via sharing or the exchange of electrons between sorbent and sorbate [64]. The calculated *q_e_* values are mostly larger for the nanocomposite NF systems and indicate that such samples particularly as UV-irradiated are able to remove up to even 50 mg·g^−1^ at the equilibrium state.

Despite the significant MB removal efficiency of the nanocomposite NFs, there is a minor fraction of MB molecules remaining on the NFs. Reusability of the system for further applications could be of importance. According to the desorption test, the nanocomposite NFs can release 33%, 22%, and 18% (under alkaline, neutral, and acidic condition, respectively) MB compared to 100% MB in the primary solution. Recalling the decomposition of a large fraction of MB molecules via the photocatalytic process, such an amount of desorbed MB molecules implies the significant regeneration ability of the NF system. At the highest pH value, a large concentration of the negatively charged hydroxyl ions in the solution can loosen the bonding between the MB molecules and the NFs surface, leading to their release. This happening is seen with a less intensity for the lower pH values.

## 4. Conclusions

The nanohybrid photocatalysts comprising polymeric NFs and inorganic NPs have proved efficient in the photodecomposition of organic water pollutants. However, the photooxidation can adversely impact the physicochemical properties of the polymer host. This aspect of utilization of the photocatalytic NPs along with the organic NFs must be taken into account and quantified to justify the nanohybridization strategy. Despite the importance of such a necessity for the industrialization of the developed nanohybrids, it has rarely been considered and studied. Here, we developed a photocatalytic NF system (TiO_2_/PES) that can effectively remove dye pollutants from water and challenged it by various thermomechanical tests to detail its performance when UV-irradiated. While photodegradation is inevitable as witnessed by the loss of thermomechanical properties, it is not so problematic that leads to discarding the samples. At the worst scenario, the UV-irradiated nanocomposite NFs show comparable thermomechanical properties with the neat PES fibers (e.g., an almost equal storage modulus of 32–35 MPa), while offering a significantly higher dye removal efficiency (95% vs. 30%). As a prospective need, the TiO_2_ NPs must be doped to enable the photodegradation of organics even under visible light, and the incorporation of sacrificial fillers that lower the degradation extent of the polymer host can be considered.

## Figures and Tables

**Table 1:** The thermal decomposition temperature (T_d_) of the PES and TiO_2_/PES nanofibrous mats.

Filling Factor	UV-Irradiation	T_d_ (°C)
**0 wt.% TiO_2_**	No	498
**5 wt.% TiO_2_**	No	509
**5 wt.% TiO_2_**	Yes	502
**8 wt.% TiO_2_**	No	522
**8 wt.% TiO_2_**	Yes	470

**Table 2:** The first-order apparent rate constant (*k*_app_) values for the photocatalytic degradation of MB.

	4 mg·L^−1^	4 mg·L^−1^	7 mg·L^−1^	7 mg·L^−1^	9 mg·L^−1^	9 mg·L^−1^
	K_app_ (min^−1^)	R^2^	K_app_ (min^−1^)	R^2^	K_app_ (min^−1^)	R^2^
**PES**	0.001	0.976	0.0005	0.923	0.0011	0.685
**UV-irr. PES**	0.0003	0.970	0.00204	0.998	0.0003	0.930
**TiO_2_/PES**	−0.0048	0.936	0.0035	0.75	0.0016	0.937
**UV-irr. TiO_2_/PES**	0.0102	0.777	0.01073	0.999	0.0147	0.999

**Table 3:** The second-order rate constant (*k*_2_) values for the photocatalytic degradation of MB.

	4 mg·L^−1^	4 mg·L^−1^	4 mg·L^−1^	7 mg·L^−1^	7 mg·L^−1^	7 mg·L^−1^	9 mg·L^−1^	9 mg·L^−1^	9 mg·L^−1^
	K_2_ (min^−1^)	q_e_ (mg·g^−1^)	R^2^	K_2_ (min^−1^)	q_e_ (mg·g^−1^)	R^2^	K_2_ (min^−1^)	q_e_ (mg·g^−1^)	R^2^
**PES**	0.003	7.52	0.979	0.001	7.57	0.778	0.0003	18.18	0.397
**UV-irr. PES**	0.004	6.06	0.999	0.00002	60.24	0.859	0.0066	14.28	0.996
**TiO_2_/PES**	0.005	10.43	0.998	0.007	30.30	0.999	0.0018	29.41	0.992
**UV-irr. TiO_2_/PES**	0.003	19.37	0.996	0.00086	36.36	0.999	0.0007	50	0.999
